# High manganese exposure decreased the risk of high triglycerides in workers: a cross-sectional study

**DOI:** 10.1186/s12889-020-09011-x

**Published:** 2020-06-05

**Authors:** Xiaoyu Luo, Zhenfang Liu, Xiaoting Ge, Sifang Huang, Yanting Zhou, Defu Li, Longman Li, Xiang Chen, Lulu Huang, Qingzhi Hou, Hong Cheng, Lili Xiao, Chaoqun Liu, Yunfeng Zou, Xiaobo Yang

**Affiliations:** 1 Department of Occupational Health and Environmental Health, School of Public Health, Guangxi Medical University, Nanning, Guangxi China; 2 Department of Hematology, The First Affiliated Hospital of Guangxi Medical University, Nanning, Guangxi China; 3 Department of Nutrition and Food Hygiene, School of Public Health, Guangxi Medical University, Nanning, Guangxi China; 4 Department of Toxicology, School of Public Health, Guangxi Medical University, Nanning, Guangxi China; 5 Guangxi Colleges and Universities Key Laboratory of Prevention and Control of Highly Prevalent Diseases, Guangxi Medical University, Nanning, Guangxi China; 6 Center for Genomic and Personalized Medicine, Guangxi Medical University, Nanning, Guangxi China

**Keywords:** Mn, Occupation, Dyslipidaemia, Triglycerides

## Abstract

**Background:**

Manganese (Mn) participates in lipid metabolism. However, the associations between Mn exposure and dyslipidaemia is unclear.

**Methods:**

This was a cross-sectional study. Data were collected from the 2017 the Mn-exposed workers healthy cohort (MEWHC). Finally, 803 occupationally Mn-exposed workers included in the study. The workers were divided into two groups. The grouping of this study was based on Mn-Time Weighted Averages (Mn-TWA). The high-exposure group included participants with Mn-TWA greater than 0.15 mg/m^3^. The low-exposure group included participants with Mn-TWA less than or equal to 0.15 mg/m^3^. Mn-TWA levels and dyslipidaemia were assessed.

**Results:**

After adjustment for seniority, sex, cigarette consumption, alcohol consumption, high-fat diet frequency, medicine intake in the past two weeks, egg intake frequency, drinking tea, WHR, and hypertension, Mn-TWA levels was negatively correlated with high triglycerides (TG) risk in workers overall (OR = 0.51; 95% CI: 0.36, 0.73; *p* <  0.01). The results of males and females were consistent (OR = 0.53; 95% CI: 0.34, 0.81; *p* <  0.01) and (OR = 0.47; 95% CI: 0.24, 0.94; *p* <  0.01), respectively. By performing interactions analyses of workers overall, we observed no significant interactions among confounders. Mn-TWA levels and pack-years on high TG risk (relative excess risk for the interactions (RERI = 2.29, 95% CI: − 2.07, 6.66), (RERI) = 2.98, 95% CI: − 2.30, 8.26). Similarly, smoking status, drinking status, high-fat diet frequency, and Waist-to-Hip Ratio (WHR) showed non-significant interactions with Mn-TWA levels on high TG risk.

**Conclusions:**

This research indicates that high Mn exposure was negatively related to high TG risk in workers.

## Background

Mn is vital for human health and is reflected in physiological metabolism [1–3]. Equally, occupational overexposure to Mn exerts neurotoxic effects [4–7]. Mn participates in lipid metabolism. However, its mechanical effects are currently unclear. Mn reduces the total antioxidant status of rats and increases brain lipid peroxidation [8, 9]. Moreover, Mn enhances cholesterol biosynthesis in the rats’ liver microsome. And stimulates farnesyl pyrophosphate synthase activity. An important synthesis pathway for many lipids in the mevalonate pathway, with mevalonate being the first branch in this pathway [10, 11]. Moreover, Mn enhances cholesterol biosynthesis in the rats’ liver microsome. And stimulates farnesyl pyrophosphate synthase activity. An important synthesis pathway for many lipids in the mevalonate pathway, with mevalonate being the first branch in this pathway [12–15].

There are few studies on Mn and lipids. A recent study showed that exposure of low-level Mn reduced serum triglyceride (TG) levels in rats [16]. Besides, epidemiological data were concentrated only on the intake of Mn. A diet study from china indicated that dietary Mn negatively correlated with hypertriglyceridaemia in males. And females’ high-density lipoprotein cholesterol (HDL-C) levels increases with Mn intake [17]. Similarly, the HDL-C levels of obese males decreased with the increased in serum Mn levels [18]. Other Chinese diet study observed that Mn intake was inversely associated with serum TG and total cholesterol (T-CHO) levels [19].

In recent decades, welding and smelting associated leaded to Mn overexposure [20]. Additionally, with the use of methyl cyclopentadienyl Mn tricarbonyl (MMT) was added to the gasoline component, resulting in increased Mn exposure in the general population [21]. Public health issues related to Mn have attracted more attention.

The MEWHC was a vertically innovative and multi-course scientific study, which began in the iron and Mn concentrator from July to October 2011 [22]. The critical overall goal of MEWHC was to explore early or long-term physical and mental health hazards, potential exposure to biomarkers, and conditions related to Mn exposure. Therefore, we carried out this study to investigate the correlations between Mn external exposure and hyperlipidemia. And we tried to explore the relationship between Mn exposure and serum lipids among Mn exposed workers.

## Methods

### Data collection

It was a cross-sectional study based on the follow-up of the 2017 MEWHC study. Detailed inclusion criteria and exclusion criteria for the cohort have been described in detail before [22, 23]. And the information collected in this cohort follow-up has been described in detail [24]. It mainly covers three types of data, including the personal information of the participants in the questionnaire, the exposure data of Mn concentrations in the workplace, collection, and storage of biological specimens and biochemical detection data. Standardized and structured questionnaires were used to collect necessary worker information. And participants were surveyed face to face by professionally trained graduate students. The information collected by the participants includes cigarette consumption, alcohol consumption, high-fat diet frequency, medicine intake in the past two weeks, egg intake frequency, drinking tea, and medical history. Standardized methods were used to measure participants’ blood pressure. The definition of hypertension adopted the latest Chinese standards [25]. Waist-to-hip ratio (WHR) was deemed high if ≥0.9 for males, and ≥ 0.85 for females (WHO, 1999). Other indicators such as cigarette and alcohol consumption were defined in detail in our previous studies [26]. Smoking 20 cigarettes a day in a year was defined as a pack-year [27]. We further divided workers’ cigarette consumption into three categories, based on the median of pack-years: non-smokers, < 18 pack-years, and ≥ 18 pack-years. According to the exclusion criteria, we excluded workers with cancer, coronary heart disease, stroke disease, or diabetes. And we excluded workers whose serum lipids were not tested due to insufficient biological samples. In the end, 22 workers were excluded from the study. A total of 803 workers participated in this study.

### Measurement of Mn levels in respirable dust

Recent researches by our team have introduced in detail the sampling and monitoring methods of Mn concentrations [24, 26]. Therefore, we briefly described the monitoring of air Mn. We have selected 20 types of jobs covering different levels of Mn exposure in the ferromanganese alloy smelter. We randomly selected three workers in each position for personal sampling. We used individual samplers to measure workers’ respiratory dust samples for three consecutive days. In the end, we collected 134 air samples. We strictly abided by China’s sampling and testing standards. The standards we adopted are as follows: “Determination of airborne dust in the workplace part 2: concentrations of respirable dust” (GBZ/T 192.2–2007); “Specifications of air sampling for hazardous substances monitoring in the workplace” (GBZ159–2004); “Ambient air and stationary source emission-determination of metals in ambient particulate matter-Inductively Coupled Plasma Mass Spectrometry (ICP-MS)” (HJ657–2013). After the digestion of the filter sample, it was measured by ICP-MS (Perkin Elmer, NexION 2000, USA). The limit of detection (LOD) of Mn was 0.076 μg/L. Based on the standard of China, PC-TWA of Mn was 0.15 mg/m^3^ (as MnO_2_). Five hundred twenty workers with Mn-TWA > 0.15 mg/m^3^ were defined as a high-exposure group. Two hundred eighty-three workers with Mn-TWA ≤ 0.15 mg/m^3^ were defined as the low-exposure group.

Smelter workers accounted for the most substantial proportion of the high exposure group. High exposure group also included ferromanganese alloy crushing operation workers, pouring crane workers, and crane workers with ferromanganese alloy raw materials. The low-exposure group mainly included circulating cooling water system operators, chemical analysts, office workers, security guards and workers in other auxiliary positions.

### Measurement of serum lipids

The determination of serum lipids has been described before [24]. The definition of serum lipids abnormality adopted the 2016 Chinese guidelines on prevention and treatment of dyslipidaemia in adults [28]. High LDL-C was defined as Low-density lipoprotein cholesterol ≥4.14 mmol/L. High TG was defined as triglyceride ≥2.26 mmol/L, high T-CHO was defined as total cholesterol ≥6.22 mmol/L, and low HDL-C was defined as high-density lipoprotein cholesterol < 1.04 mmol/L. Dyslipidaemia can further develop into cardiovascular disease [29]. Dyslipidaemia guidelines suggested an LDL-C target should be set according to individual ASCVD risk. The Chinese guidelines suggested that LDL-C target should be set based on an individual’s ASCVD risk levels. The personal ASCVD risk level was evaluated to age, sex, Body Mass Index (BMI), hypertension history, and cigarette consumption [28].

### Statistical analysis

The Mann-Whitney U test was used to compare serum lipids levels in different groups. We used logistic regression models to estimate Mn exposure levels and the risk of varying serum lipid abnormalities. Also, there was a high correlation between age and years of work. Only the working years were adjusted in the models. Corrected confounders included sex, cigarette consumption, alcohol consumption, high-fat diet frequency, medicine intake in the past two weeks, egg intake frequency, drinking tea, WHR, and hypertension.

We also conducted a hierarchical analysis. Besides, biological interactions between confounding factors were also evaluated. Rothman et al. suggested that studies should pay attention to epidemiological interactions or additive interactions. The method assessed whether the combined effect of exposure to two factors was higher than the sum of their independent effects.

The authors proposed the use of relative excess risk for interactions (RERI) in assessing additive interactions. Rothman et al. explained detailed RERI explanations and calculation methods in the article [30–32]. The interaction between Mn-TWA levels and confounders were evaluated. Confounders included cigarette consumption (smoking status and pack-years), alcohol consumption, high-fat diet frequency, medicine intake in the past two weeks, and WHR. The analysis software we use is R (version 3.4) and SPSS (version 19.0) A two-sided, *p* <  0.05 was considered statistically significant.

## Results

In our participants, The median (IQR) ages were 41.75(36.58,46.92) and 45.42 (41.27,49.08) years for low, and high exposure groups, respectively. The median seniority was 18.92 years. And no significant difference was observed in seniority between two groups (*p* = 0.07). The proportion of males in the two groups was 57.6 and 72.5%, respectively. The ratio of males who consumed cigarettes was higher in high-exposure group, at 26.2% (*p* <  0.01). And 10.6% of consumed cigarettes in low exposure group. Similarly, the proportion of ≥18 pack-years was higher in the high exposure group, and the rates were 29.4, 14.5%, respectively (*p* <  0.01). Alcohol consumption at the high exposure was higher, at 32.7% (*p* <  0.01). Low-exposure was 23.0%. WHR, hypertension, drinking tea, and medicine intake in the past two weeks were not different in two groups (*p*>0.05) (Table 1). High TG (≥2.3 mmol / L), high T-CHO (≥6.1 mmol / L), high LDL-C (≥4.10 mmol / L) and low HDL-C (< 1.04 mmol / L) were 25.5, 15.7, 6.8 and 3.1%. According to the individual’s ASCVD risk, the overall goal of LDL-lowering was set, and the incidence rate is 27.4%. The incidence of high TG in the low-exposure group was higher than that in the high-exposure group, which were 30.7 and 22.7%, respectively (*p* <  0.01)) (Table 2).

**Table 1:** Demographic characteristics of the manganese-exposed workers from MEHWC

Variables	Total (*n* = 803)	Low exposure group (*n* = 283)	High exposure group (*n* = 520)	*p* –Value*
Age (years)	44.25 (39.50,48.42)	41.75 (36.58,46.92)	45.42 (41.27,49.08)	< 0.01
Seniority (years)				0.07
< 18.92	402 (50.1)	154 (54.4)	248 (47.7)	
≥18.92	401 (49.9)	129 (45.6)	272 (52.3)	
BMI (kg/m^2^)				< 0.01
< 24	317 (39.5)	131 (46.3)	186 (35.8)	
≥24	486 (60.5)	152 (53.7)	334 (64.2)	
Gender				< 0.01
Male	540 (67.2)	163 (57.6)	377 (72.5)	
Female	263 (32.8)	120 (42.4)	143 (27.5)	
Race				0.65
Han	361 (45.0)	128 (45.2)	233 (44.8)	
Zhuang	409 (50.9)	141 (49.8)	268 (51.5)	
Other race	33 (4.1)	14 (4.9)	19 (3.7)	
Education level				< 0.01
Middle school or below	257 (32.0)	39 (13.8)	218 (41.9)	
High school	367 (45.7)	103 (36.4)	264 (50.8)	
Junior college or above	179 (22.3)	141 (49.8)	38 (7.3)	
Smoking status				< 0.01
Nonsmoker	476 (59.3)	208 (73.5)	268 (51.5)	
Former smokers	161 (20.0)	45 (15.9)	116 (22.3)	
Current smokers	166 (20.7)	30 (10.6)	136 (26.2)	
Pack-years^a^				< 0.01
Nonsmoker	431 (53.7)	193 (68.2)	238 (45.8)	
< 18 years (low)	178 (22.2)	49 (17.3)	129 (24.8)	
≥18 years (high)	194 (24.1)	41 (14.5)	153 (29.4)	
Drinking status				< 0.01
Former/never drinker	568 (70.7)	218 (77.0)	350 (67.3)	
Current drinker	235 (29.3)	65 (23.0)	170 (32.7)	
Hypertension				0.23
Yes	251 (31.3)	81 (28.6)	170 (32.7)	
No	552 (68.7)	202 (71.4)	350 (67.3)	
WHR				0.69
High	294 (36.6)	193 (37.1)	101 (35.7)	
Normal	509 (63.4)	327 (62.9)	182 (64.3)	
Medicine intake in the past two weeks				0.85
Yes	283 (35.2)	69 (34.7)	214 (35.4)	
No	520 (64.8)	130 (65.3)	390 (64.6)	
Drinking tea				0.99
Yes	488 (60.8)	316 (60.8)	172 (60.8)	
No	315 (39.2)	204 (39.2)	111 (39.2)	
High-fat diet frequency				< 0.01
< 3times/week	640 (79.7)	242 (85.5)	398 (76.5)	
≥3times/week	163 (20.3)	41 (14.5)	122 (23.5)	
Egg intake frequency				< 0.01
< 3times/week	602 (75.0)	408 (78.5)	194 (68.6)	
≥3times/week	201 (25.0)	112 (21.5)	89 (31.4)	

*Mn-TWA* Mn-Time Weighted Average, Low exposure group, Mn-TWA ≤0.15 mg/m^3^; High exposure group, Mn-TWA > 0.15 mg/m^3^; MEHWC, Manganese-exposed workers healthy cohort; WHR, Body Mass Index; Data were presented as median (25th, 75th) or *n* (%)

*p –Value were derived from Mann-Whitney U tests for continuous variables according to the data distribution, and chi-square test for the categorical variables

pack-years ^a^: A pack-year was defined as 20 cigarettes smoked every day for 1 year. We further categorized participants’ smoking status into three groups on the basis of median pack-years: nonsmokers, < 18 pack-years, and ≥ 18 pack-years

**Table 2:** Prevalence of different forms of dyslipidaemia among participants from MEHWC

Variables	Total(n = 803)	Low exposure group(*n* = 283)	High exposure group(*n* = 520)	*p* –Value*
Triglycerides				0.01
≥ 2.3 mmol/L	205 (25.5)	87 (30.7)	118 (22.7)	
< 2.3 mmol/L	598 (74.5)	196 (69.3)	402 (77.3)	
Total cholesterol				0.18
≥ 6.2 mmol/L	126 (15.7)	51 (18.0)	75 (14.4)	
< 6.2 mmol/L	677 (84.3)	232 (82.0)	445 (85.6)	
LDL-C				0.44
≥ 4.1 mmol/L	55 (6.8)	22 (7.8)	33 (6.3)	
< 4.1 mmol/L	748 (93.2)	261 (92.2)	487 (93.7)	
HDL-C				0.61
< 1.0 mmol/L	25 (3.1)	10 (3.5)	15 (2.9)	
≥ 1.0 mmol/L	778 (96.9)	273 (96.5)	505 (97.1)	
No achieving LDL-lowering targets ^b^				0.57
Yes	220 (27.4)	81 (28.6)	139 (26.7)	
No	583 (72.6)	202 (71.4)	381 (73.3)	

*Mn-TWA*, Mn-Time Weighted Average; Low exposure group, Mn-TWA ≤0.15 mg/m^3^; High exposure group, Mn-TWA > 0.15 mg/m^3^; LDL-C, Low-density lipoprotein cholesterol. HDL-C, High-density lipoprotein cholesterol; MEHWC, Manganese-exposed workers healthy cohort; No achieving LDL-lowering targets ^b^, According to the Chinese guideline-2016 Chinese Guideline for the Management of dyslipidaemia in Adults [28], LDL-lowering targets were set according to individual ASCVD risk levels. Adjusted by age, gender, WHR, history of hypertension, and smoking status

^*^p –Value were derived from chi-square test

**Fig. 1:**
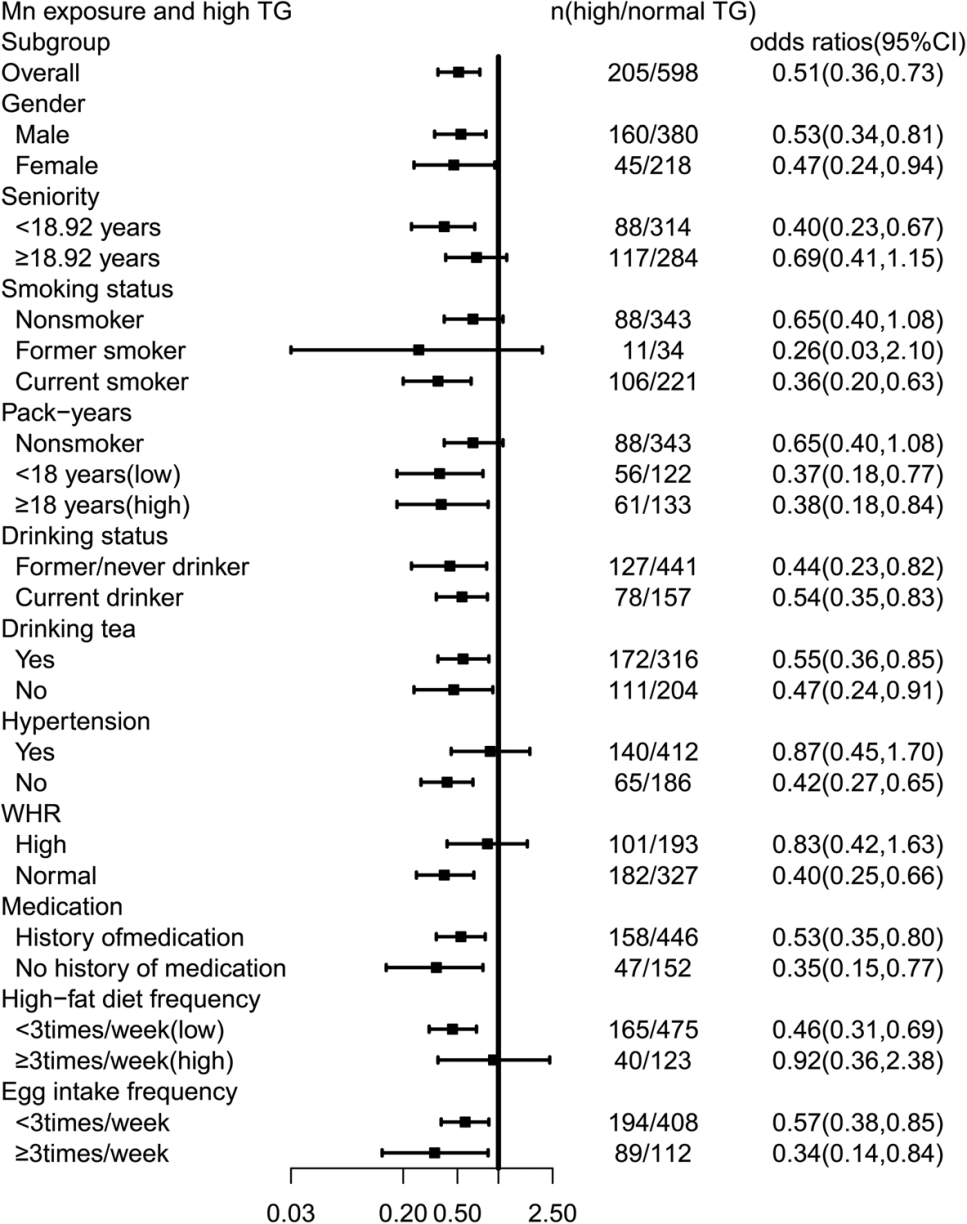
Adjusted ORs for Mn-TWA levels associated with high TG risk in subgroups. Logistic regression models was used for analysis, with high TG as the dependent variable and Mn-TWA levels (categorical variable) as the independent variable. We set subgroups according to gender, seniority, smoking status, pack years, drinking status, hypertension, egg intake frequency, drinking tea, medicine intake in the past two weeks, and WHR. Seniority was divided into two groups by median, and other variables were adjusted. When participants were males, or current smokers, or smoking ≥18 pack-years, seniority < 18.92 years, or non-hypertension, or high-fat diet frequency less than 3 times per week, or WHR, Mn-TWA levels showed negative associations with high TG risk

Adjusted for potential confounding factors as sex, seniority, WHR, high blood pressure, drug intake in the past half month, high-fat diet consumption, egg intake frequency, drinking tea, smoking and drinking status, high TG risk significantly decreased in high exposure group (OR = 0.66; 95% CI: 0.48, 0.92; *p* <  0.01), and consistent negative correlation was found in males (OR = 0.53; 95% CI: 0.34, 0.81; *p* <  0.01) and females (OR = 0.47; 95% CI: 0.24, 0.94; *p* <  0.01). Similarly, the results negative correlation between high Mn-TWA levels and high TG risk were found among subgroups current smokers (OR = 0.36; 95% CI: 0.20, 0.63), and <  18 pack-years group (OR = 0.37; 95% CI: 0.18, 0.77), ≥18 pack-years group (OR = 0.38; 95% CI: 0.18, 0.84), seniority < 18.92 years group (OR = 0.40; 95% CI: 0.23, 0.67), non-hypertension group (OR = 0.42; 95% CI: 0.27, 0.65), high-fat diet frequency < 3 times per week group (OR = 0.46; 95% CI: 0.31, 0.69), and normal WHR group (OR = 0.40; 95% CI: 0.25, 0.66) (Table 3, Fig. 1).

**Table 3:** Adjusted odds ratios [95% confidence interval (CI)] for different forms of dyslipidaemia according to Mn-TWA levels in MEHWC

dyslipidaemia	Model 1^*^	*p* –Value	Model 2^**^	*p* –Value
	OR(95% CI)		OR(95% CI)	
No achieving LDL-lowering targets	0.91 (0.66,1.26)	0.48	0.77 (0.54,1.09)	0.14
High LDL-C	0.80 (0.46,1.41)	0.80	0.70 (0.39,1.28)	0.25
High TG	0.66 (0.48,0.92)	0.01	0.51 (0.36,0.73)	< 0.01
High T-CHO	0.77 (0.52,1.13)	0.18	0.71 (0.47,1.08)	0.11
Low HDL-C	0.81 (0.36,1.83)	0.61	0.55 (0.23,1.30)	0.17

Logistic regression models was used for analysis, with different forms of dyslipidaemia as the dependent variable and Mn-TWA levels (categorical variable) as the independent variable. No achieving LDL-lowering targets, low-density lipoprotein cholesterol targets were set according to individual ASCVD risk, and adjusted for the variables as drug status in the past 2 weeks, and alcohol intake status. According to the Chinese guideline-2016 Chinese Guideline for the Management of dyslipidaemia in Adults [28], high LDL-C was defined as Low-density lipoprotein cholesterol ≥4.14 mmol/L, high TG was defined as triglycerides ≥2.3 mmol/L, high T-CHO was defined as total cholesterol ≥6.2 mmol/L, and low HDL-C was defined as High-density lipoprotein cholesterol < 1.0 mmol/L.

^*^Model 1: Without adjusting covariates

^**^Model 2: Adjusted for the variables as gender, seniority, WHR, hypertension, medicine intake in the past two weeks, high-fat diet frequency, egg intake frequency, drinking tea, smoking status, and drinking status

In the analysis of interaction, we did not observe that there was a significant cumulative scale interaction between Mn-TWA levels and cigarette consumption status or the pack-years of high TG risk (relative excess risk for the interactions for cigarette consumption (RERI = 2.29, 95% CI: − 2.07, 6.66), (RERI) = − 2.98, 95% CI: − 1.88, 7.85) for pack-years, respectively. Research showed that the mutual harm of high-quality Mn-TWA exposure and current smoking or previous smoking did not exceed the total number of their hazards, and consistent results were found in ≥18 pack-years or <  18 pack-years. In the same way, no obvious interactions between Mn-TWA levels and alcohol consumption, the frequency of high-fat diet, and the risk of high TG **(**Table 4**).**

**Table 4:** Adjusted odds ratios [95% confidence interval (CI)] for high TG according to the combined exposure Mn-TWA levels with categories of smoking status, pack-years, drinking status High-fat diet, and WHR in male workers

Variables	n (high /normal TG)	Low exposure	High exposure	Relative excess risk due to interactions (RERI)^**^ (95%CI)
		OR^*^(95% CI)	OR^*^(95% CI)	
Smoking status				
nonsmokers	88/343	1.00	0.71 (0.44,1.16)	
Former smokers	11/34	1.19 (0.37,3.80)	0.45 (0.16,1.33)	2.29 (−2.07,6.66)
Current smokers	106/221	2.20 (1.14,4.23)	0.75 (0.43,1.28)	2.98 (−1.88,7.85)
Pack years				
nonsmokers	88/343	1.00	0.71 (0.44,1.16)	
< 18 years (low)	56/122	2.09 (0.99,4.39)	0.77 (0.42,1.41)	2.29 (−2.07,6.66)
≥18 years (high)	61/133	1.83 (0.83,4.02)	0.66 (0.36,1.19)	2.98 (−2.30,8.26)
Drinking status				-0.56 (−1.80,0.69)
Former/never drinker	127/441	1.00	0.55 (0.36,0.84)	
Current drinker	78/157	1.37 (0.72,2.59)	0.60 (0.35,1.02)	
High-fat diet frequency				5.82 (−20.28,31.92)
<3 times/week (low)	165/475	1.00	0.49 (0.33,0.72)	
≥3 times/week (high)	40/123	0.79 (0.35,1.76)	0.51 (0.30,0.87)	
Hypertension				2.26 (−2.03,6.65)
Yes	65/186	1.00	0.40 (0.26,0.62)	
No	140/412	0.63 (0.34,1.15)	0.54 (0.33,0.88)	
WHR				14.61 (−62.23,91.45)
Normal	103/91	1.00	0.43 (0.27,0.69)	
High	102/407	1.40 (0.80,2.47)	0.88 (0.54,1.45)	

Low exposure group, Mn-TWA ≤0.15 mg/m^3^; High exposure group, Mn-TWA > 0.15 mg/m^3^. pack-years, A pack-year was defined as 20 cigarettes smoked every day for 1 year [27]. We further categorized participants’ smoking status into three groups on the basis of median pack-years: nonsmokers, < 18 pack-years, and ≥ 18 pack-years

^*^OR: In our cohort, only participants were males, Mn-TWA levels showed stronger negative associations with high TG risk. And OR across the combined exposure of Mn-TWA levels and the other risks factors of dyslipidaemia were obtained in logistic regression models in male. Adjusted for the variables as gender, seniority, smoking status, pack-years, drinking status, hypertension, and medicine status in the past two weeks, egg intake frequency, drinking tea and WHR; Combined categories variables did not be adjusted

**RERI: We assessed the presence of interactions between exposure Mn-TWA levels and smoking status, pack-years, drinking status, High-fat diet, and WHR by testing whether the joint effect from exposure to both factors was greater than the sum of their independent effects. When the relative excess risk for interaction > 0, there is an additive scale interactions between the two risk factors, and the 95% confidence interval is positive and does not contain 0. Otherwise, there was no interactions, and RERI = 0 indicates exact additivity and there is no additive scale interactions

## Discussion

The associations between Mn exposure and dyslipidaemia in occupational workers were first discussed. The results showed that workers’ exposure to higher Mn-TWA levels was associated with lower TG risk. And there was no interaction with confounders. Most of the researches concentrated on the intake of Mn in the diet. One clinical study showed that when 14 adults filled the gluconic acid in the diet, Mn reduced body fat by increasing the body fat metabolism of excreta [33]. Another clinical study for 7 young men showed that adequate intake Mn could reduce blood carbohydrate levels [34]. One study on the diet of 2111 Chinese participants found that male’s Mn intake was inversely proportional to hypertriglyceriduria. And that females’ HDL-C concentrations increased with Mn intake [17]. One Chinese study on the absorption of polymetallic diets for 258 healthy males and females found that the consumption of Mn was negatively proportional to serum TG and T-CHO [19]. In this study, Mn exposure of workers was inversely proportional to serum TG. However, we did not observe correlations between Mn exposure and serum T-CHO, HDL-C, LDL-C, and LDL-lowering targets. Previous researches have already confirmed the critical efficacy of Mn in TG regulation. However, our participants were occupationally touched to Mn. Therefore, it was not appropriate to compare dietary Mn intake with the concentrations of occupational Mn exposure levels of our workers. It was essential to study the mechanism of Mn′s involvement in lipids metabolism and to assess the toxic doses of Mn to dyslipidaemia. It was essential to consider the mechanism of Mn′s involvement in lipids metabolism and to determine the toxic doses of Mn to dyslipidaemia. Besides, mammalian models must be established to show that inhalation Mn exposure concentrations were closer to the occupationally touched to Mn.

Mn can enter peripheral blood through intestinal absorption and olfactory channels. The steady-state Mn ions in the peripheral blood were further absorbed and metabolized by the liver. In contrast, excess Mn (in the form of Mn2+ is primarily excreted from the liver into the intestine, along with bile [35–38]. Previously published studies have shown that Mn metabolism was related to lipid peroxidation [39–41]. Also, studies have shown that Mn has participated in lipid metabolism through lipid synthesis [12–15]. Thus the influence of Mn in lipid mechanisms is equivocal.

In terms of lipid synthesis, two pathways exist for TG synthesis in the liver. One of the mechanisms was the entry of exogenous fatty acids into hepatocytes, which are then esterified to synthesize TG. TG can also pass on the de novo body fat production (DNL) pathway. Eventually, TG is placed in a storage tank or secretion tank. TG can also pass on the de novo body fat production (DNL) pathway. Eventually, TG is placed in a storage tank or secretion tank [42–45]. Acetyl-CoA carboxylase (ACC) was an important metal catalyst for the production of the Novo DNL acids (necessary phosphatases). Phosphatase was a necessary auxiliary enzyme to active ACC. And phosphatase relied on Mn2 + activation and dephosphorylation to participate in ACC activity [43, 46]. Therefore, Mn 2+ plays a crucial role in the synthesis of TG in the liver. The allosteric inhibition of liver ACC significantly reduces hepatic TG concentrations and increased plasma TG levels [47, 48]. It is speculated that the inhibition of ACC is the mechanism of manganese-induced hypertriglyceridemia [49]. We hypothesized that higher levels of Mn2+ were stored in the livers of workers exposed to higher Mn levels. And ACC was more likely to be activated in the liver. Eventually, TG levels in the liver may be higher, while TG levels in serum are reduce.

Gender is a common factor affecting Mn absorption. Previous studies have found that females have higher Mn absorption capabilities. And Males were found to have lower levels of Mn in their blood than females [50–53]. However, males are reported to be more prone to TG, lipid abnormalities, and metabolic diseases [54–56]. TG metabolism is regulated by endogenous estrogen and androgen [57]. Currently, several studies have observed that in hepatocyte-specific ERa-knock-out mice, estrogen cannot make liver fatty degeneration. This result suggests that estrogen directly acts on the liver via the Estrogen Receptor alpha (ERa), thereby decreasing TG [58–60]. In additionally, to cope with obesity, both males and females increase the flow of fatty acids into the peripheral blood. Visceral or visceral chamber fat contributes more to liver fatty acid delivery, than subcutaneous fat [46]. The fatty acids that are absorbed into the liver are assembled into TG. And then wrapped up in TG rich very low-density lipoprotein (VLDL) particles and expelled from the liver [61, 62]. Other studies have observed that females can produce more TGDL-rich VLDL particles, and these particles help reduce overall blood TG levels.

When the body ingests food, TG circulates in the form of chylomicrons containing apolipoprotein 48. Study through short-term and long-term high-fat feeding found that females can better clear diet-related TG [63–65]. Consistent with previous research results, the proportion of females with high TG was indeed lower in our study subjects, and that high TG was observed in males. But the high TG risk is not different between males and females workers after exposure to Mn. We speculate that we may have corrected WHR factors in the statistical analysis, to avoid confounding effects caused by sex hormones. Therefore, our research can reflect that manganese is involved in TG metabolism.

In our study, smoking and drinking rates were higher than the general population, our smoking and drinking rates were 40.7 and 28.4%, respectively, were wherein a survey of 163,641 Chinese adults between 2013 and 2014, the rates were 24.4 and 8.7%, respectively. Guidelines on the treatment of blood cholesterol to reduce atherosclerosis by The American Heart Associations (AHA, 2013) have indicated that smoking was an independent risk behaviour for dyslipidaemia. That small amounts of alcohol could raise TG levels further [29]. Previous studies have shown that WHR and high-fat diet are significantly and positively correlated with high TG levels [66–70]. However, from interactions analyses, we observed no interactions between Mn-TWA levels and smoking effects (both smoking status and pack-years), drinking status, high-fat diet, and the WHR on high TG risk. Although not statistically significant, regardless of whether the population’s high-fat diet frequency was high or low, we can observe a negative correlation between Mn-TWA levels exposure and high TG risk. This result suggests that the intensity of Mn exposure to decrease high TG risk was greater than that of a high-fat diet. Further investigations are required to confirm these findings.

This is the first study to examine the relationship between Mn exposure and dyslipidaemia in occupational workers. We comprehensively carried out a full range of accurate measurements and analysis of the risk sources and potential risks related to lipid metabolism. And we will further conduct follow-up the cohort to evaluate the risk of hyperlipidemia exposed to Mn. There were some limitations to our study. Firstly, non-Mn exposed individuals were not included as controls. Therefore, the confounding effect of regional diet mix, labor efficiency, genetic inheritance, and environmental hazards cannot be ruled out. Our data does not accurately reflect Mn cumulative exposure indices (Mn-CEI). So there is no way to comprehensively discuss the relationships between long-term Mn cumulative exposure and dyslipidaemia.

## Conclusions

This study observed an inverse correlation between workers’ high TG and Mn exposure levels. We expect larger prospective studies to confirm the association between Mn exposure and dyslipidaemia.

## Data Availability

The data and material are available upon reasonable request from the corresponding author. E-mail: yxbo21021@163.com.

## Abbreviations

MMT: Methyl cyclopentadienyl manganese tricarbonyl
TWA: Time-weighted Average
TG: Triglycerides
T-CHO: Total Cholesterol
LDL-C: Low-density lipoprotein cholesterol
HDL-C: High-density lipoprotein cholesterol
WHR: Waist-to-Hip Ratio
RERI: Relative excess risk for the interactions
ICP-MS: Inductively Coupled Plasma Mass Spectrometry
MEWHC: Manganese-exposed Workers Healthy Cohort
QC: Quality control
PC-TWA: Permissible concentrations-time weighted average
LOD: Limit of detection
DNL: De novo lipogenesis
VLDL: Very Low density lipoprotein
Mn-CEI: Manganese-cumulative exposure index
